# Sleep differences in the UK between 1974 and 2015: Insights from detailed time diaries

**DOI:** 10.1111/jsr.12753

**Published:** 2018-09-10

**Authors:** Juana Lamote de Grignon Pérez, Jonathan Gershuny, Russell Foster, Maarten De Vos

**Affiliations:** ^1^ Centre for Time Use Research and Nuffield Department of Clinical Neuroscience Oxford University Oxford UK; ^2^ Department of Sociology Oxford University Oxford UK; ^3^ Nuffield Department of Clinical Neuroscience Oxford University Oxford UK; ^4^ Department of Engineering Oxford University Oxford UK

**Keywords:** change, deprivation, jetlag, sleep, time use

## Abstract

It is often stated that sleep deprivation is on the rise, with work suggested as a main cause. However, the evidence for increasing sleep deprivation comes from surveys using habitual sleep questions. An alternative source of information regarding sleep behaviour is time‐use studies. This paper investigates changes in sleep time in the UK using the two British time‐use studies that allow measuring “time in bed not asleep” separately from “actual sleep time”. Based upon the studies presented here, people in the UK sleep today 43 min more than they did in the 1970s because they go to bed earlier (~30 min) and they wake up later (~15 min). The change in sleep duration is driven by night sleep and it is homogeneously distributed across the week. The former results apply to men and women alike, and to individuals of all ages and employment status, including employed individuals, the presumed major victims of the sleep deprivation epidemic and the 24/7 society. In fact, employed individuals have experienced a reduction in short sleeping of almost 4 percentage points, from 14.9% to 11.0%. There has also been a reduction of 15 percentage points in the amount of conflict between workers work time and their sleep time, as measured by the proportion of workers that do some work within their “ideal sleep window” (as defined by their own chronotype).

## 1 INTRODUCTION

There is a view that sleep has declined over recent decades, and that sleep deprivation has reached epidemic levels (Roenneberg, 2013; Van Cauter & Knutson, 2008), with jobs most often blamed for this (Chatzitheochari & Arber, 2009; Drake, Roehrs, Richardson, Walsh, & Roth, 2004). Furthermore, there is a growing body of epidemiological literature showing that insufficient sleep and the disruption of circadian rhythms are associated with several health problems, such as obesity (Roenneberg, 2012) or type 2 diabetes (Chaput, Despres, Bouchard, & Tremblay, 2009) among others. This has led to public concern (Colten & Altevogt, 2006; Roenneberg, 2013).

Although there is clear physiological evidence that sleep disruption can predispose individuals to a variety of health problems, it is unclear whether sleep deprivation is a *growing* problem. The current evidence for a sleep deprivation epidemic is based mostly upon studies from the USA, and from answers to questions such as “On average, how many hours of sleep do you get in a 24‐hr period?” (e.g. the National Sleep Foundation Polls). These questions have been shown to compare poorly with actigraphy (Girschik, Fritschi, Heyworth, & Waters, 2012; Lauderdale, Knutson, Yan, Liu, & Rathouz, 2008). Time‐use diaries instead are perhaps more likely to produce answers closer to the truth because individuals are not asked to guess about their sleep, in isolation, but to recall the whole day. Covering all 24 hr of the day makes biases in estimating sleep time less likely because “overestimating” one activity means necessarily having to “reduce” at least one other time estimate so that all activities add up to 24 hr. Furthermore, because there is no emphasis on any particular activity, context effect biases are unlikely. Not surprisingly, diaries have been found to be accurate when compared with camera recordings (Gershuny et al., 2017). Hence, in the absence of nationally representative samples of objective sleep measures for several points in time, diaries may provide the best option to study sleep changes in the population.

Current diary evidence suggests time in bed has increased or stayed the same in most places (Bin, Marshall, & Glozier, 2012, 2013 ; Hoyos, Glozier, & Marshall, 2015). However, this diary evidence cannot really address the sleep deprivation epidemic proposition because it does not measure “sleep time” but “time in bed.” An increase in time in bed could be hiding a reduction in actual sleep time offset by an increase in being awake in bed. This limitation has been acknowledged previously (Chatzitheochari & Arber, 2009; Hoyos et al., 2015; Leech, 2017) but never addressed in practice.

The aim of this paper is to go beyond time in bed and investigate sleep estimates for the UK. This is, to our knowledge, the first study that builds a time series of sleep time based upon diary evidence for any country. This is possible thanks to two surveys that include activity codes to track “time in bed not asleep”: the time‐use study carried out by the British Broadcasting Corporation (BBC) in 1974/75, and the most recent national time‐use study of 2014/15. This paper will also discuss the changes in social jetlag over the same time span and the interaction between work time and sleep.

## 2 MATERIALS AND METHODS

### 2.1 Data

**Figure 1:**
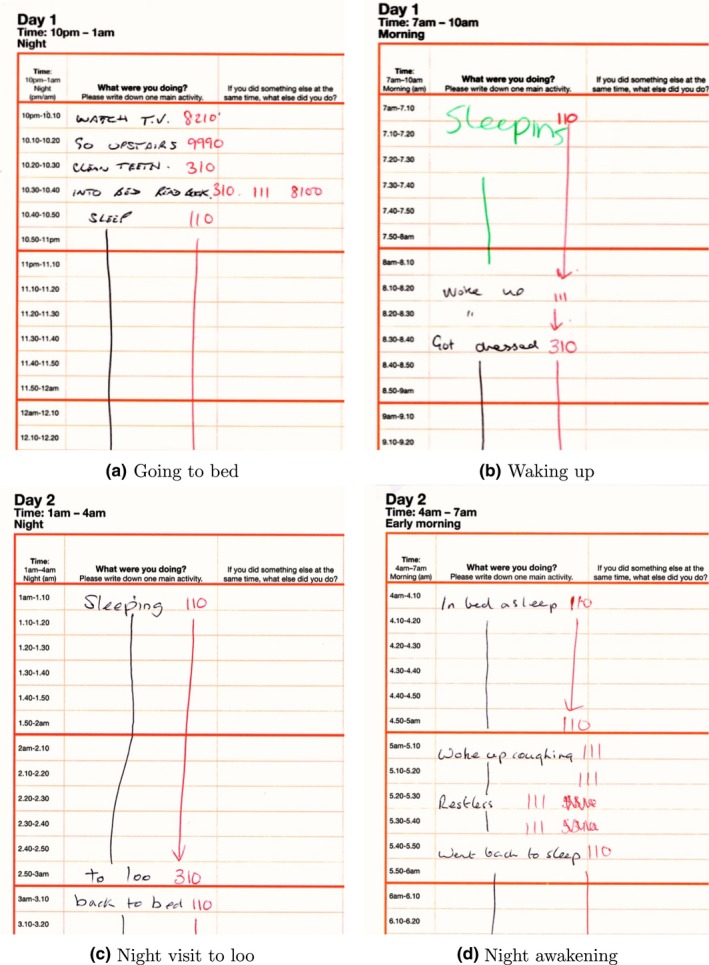
The recording of sleep in time‐use diaries. Four examples from the 2014/15 UK Time use Survey. In order to make the examples readable the figure focus is on the relevant part of the diary page: the time and the activity columns, and the moment of the day that contains sleep. The black font is the text written by the diarists and the text in green and red are notes added by coders. In red, coders write the code that corresponds to each activity; the green pen is used for other notes. In example (a) the diarist writes “INTO BED READ BOOK,” and the coder writes 310, 111 and 8100; 310 is a personal care code used whenever the diarist reports going to bed, 111 means the diarist is in bed but not asleep, and the code 8100 registers time reading. The next activity that the diarist reports is “SLEEP,” coded as 110

This paper used three nationally representative time studies for the UK from 1974/75, 2000/01 and 2014/15. The three surveys asked participants to complete diaries like the ones shown in Figure 1 and at a later stage the free‐text activity descriptions would be converted into numerical codes. The main analysis focused on the 1974/75 and 2014/15 surveys because only those identify “time in bed not asleep” separately from “actual sleep” time.

There are some important methodological differences across the surveys, which have been accounted for in the analysis (see Table 1). The difference in the number of days covered by each survey was corrected with the use of weights (in the two most recent surveys, weekdays are given 2.5 times more weight than weekends, given the oversampling of weekends). The difference in the duration of the time interval affected only the precision of the estimates. Diaries measure the time spent on activities, with errors due to fixed‐duration time intervals. This error has been shown to be Gaussian distributed and unbiased (Gershuny et al., 2017). Even if the variance of the error distribution is larger in 1974/75 than in 2014/15 because of the longer interval, both estimates are unbiased.

**Table 1:** Sample demographics and key survey features

	Survey year		
	1974/75	2000/01	2014/15
	Mean and *SD*	Mean and *SD*	Mean and *SD*
Age (years)	46.3 (17.3)	46.7 (17.3)	48.3 (18.1)
Male (%)	47.7	47.2	47.6
Employed (%)	60.0	57.8	55.0
Inactive (%)	20.5	23.1	22.0
Retired (%)	19.5	19.1	23.0
	
No. diaries	14,725	16,566	14,197
No. individuals	2,568	8,340	7,126
No. diaries per person	7 (1 week)	2 (1 weekday, 1 weekend)	2 (1 weekday, 1 weekend)
Hour coverage	05:00−02:00	04:00−04:00	04:00−04:00
Interval duration	30 min	10 min	10 min
Response rate	61%	45%	45%

Note

The analysis is restricted to the adult population. Unemployed individuals are included in the inactive as in the rest of the manuscript. More information on each survey can be found at: https://www.timeuse.org/mtus/surveys.

Another methodological difference across surveys is the hours of the night missing from the 1974/75 survey, unlike modern surveys that cover the 24 hr of the day. The BBC had no interest in what people did in the hours when no content was being broadcast (between 02:00 and 05:00 hours). A non‐controversial imputation procedure was carried out and its details can be found in Supporting information, Appendix S1.

### 2.2 Analysis

The first part of the analysis describes the changes in sleep time and social jetlag, and the second part assesses the interactions between work and sleep, to investigate if jobs were putting increasing pressure on our sleep. *Sleep duration* is defined as any sleep time in a 24‐hr period: it includes naps and excludes non‐sleeping in bed, such as time spent trying to get to sleep, time in bed after waking up and night awakenings. Both average sleep duration and the prevalence of short and long sleepers was assessed. Changes in sleep timing were also analysed. For reference, the changes in *time in bed*are also provided.

Societal determination of work time, and sometimes also of free time, interferes with individual sleep preferences. Wittmann, Dinich, Merrow, and Roenneberg (2006) introduce the term “social jetlag” to refer to this conflict and propose its measurement as the absolute difference between midsleep on workdays and midsleep on free days. Later, Jankowski (2017) suggested a correction to the formula in order to remove the sleep‐debt effect and capture only the effects of the biological/social time misalignment, which we follow in this paper. Such correction involves using different formulas for different sleep types, as shown below.

(1) SJL=|Onset on free days - Onset on work days|

(2) SJL=|Offset on free - Offset on work days|

(3) SJL=|Midsleep on free days - Midsleep on work days|

Equation (1) is used for individuals with longer sleep and later (or equal) sleep onset on free days compared with workdays, Equation (2) for individuals with longer sleep and earlier (or equal) sleep offset on workdays compared with free days, and Equation (3) for other sleep patterns. Weekdays and weekends are used to estimate jetlag obtained for the entire population and not just for individuals in employment.

Social jetlag can be seen as an attempt to measure conflict between the internal clock and socially determined schedules imposed on the individual, through an indirect examination of sleep inequality across the week. In this paper we propose also a direct measure of that conflict, defined as the amount of work within each individual's ideal sleep window. Each individual's sleep window is defined as the 10 hr surrounding midsleep on free days (chronotype). Work time information is obtained from the diaries. Then, an individual whose midsleep on free days happens at 04:00 hours has an ideal sleep window that starts at 23:00 hours and ends at 09:00 hours. If that person started work at 08:00 hours, the work–sleep conflict would be equal to 1 hr. We chose a 10‐hr window as that allowed for an 8‐hr sleep plus 2 hr of personal care, but the results were robust to changes in window size.

All estimates were obtained for the sample as a whole, as well as for different population subgroups, such as employment status, age (18–34, 35–64, 65+ years) and gender. Design weights and non‐response weights were applied to ensure that the results represent the population of the UK.

## 3 RESULTS

### 3.1 Sleep duration and sleep timing

**Figure 2:**
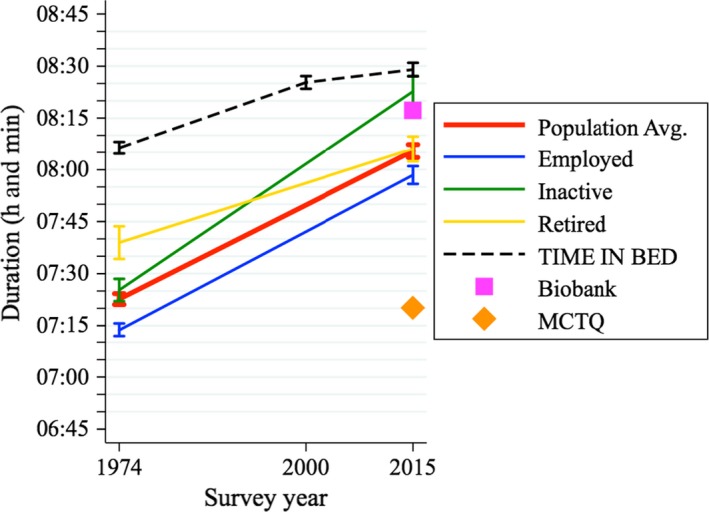
Average sleep duration and time in bed in the UK between 1974 and 2015 across employment status. *Sleep duration*is defined as any sleep time in a 24‐hr period; it includes naps and excludes non‐sleeping in bed, such as time spent trying to get to sleep, time in bed after waking up and night awakenings. *Time in bed*includes non‐sleeping. 95% confidence intervals are also shown. The changes are driven by night sleep as day sleep increases only by less than 2 min. The “inactive” includes the unemployed, who were too few to be analysed on their own. The main results come from the UK time‐use surveys but also include two sleep estimates from two other sources: the Munich Chronotype Questionnaire (MCTQ) (Till Roenneberg, personal communication in March 2018) and UK Biobank (from Willetts et al., 2017, who analysed a subsample of the Biobank study, including 103 712 participants who agreed to wear a wrist‐worn accelerometer for a 7‐day period between 2013 and 2015). The Biobank sample resembles the UK population aged 47+ years, with a “healthy volunteer” effect (Fry et al., 2017). The MCTQ is an online survey filled in by volunteers. The estimate shown here is the mean value of a sex‐and‐age normalised sleep measure for the UK entries, 21 519 individuals. The mean age is 35.7 years and 58.2% are female. Thus, the MCTQ sample is younger and has more women than the UK population.

In the UK, people slept on average 43 min more in 2015 than they did in 1974 (*p*‐value < 0.001). Average sleep duration went from 7 hr and 23 min in the 1970s, to 8 hr and 6 min in 2015 (see Figure 2). The increase was not driven by a particular sociodemographic group because all the groups examined (sex, age or employment status) experienced an increase in sleep duration. The largest increase in sleep duration was experienced by the inactive, who slept almost 1 hr more in 2015 than they did in 1974. The employed follow with 45 min and the retired are the group that increased their sleep the least (by 27 min) (all *p*‐values < 0.001). Table S1 (in Supporting information, Appendix S3) shows that there are also important differences across age groups, with younger individuals experiencing greater increases in sleep duration than older ones. Men and women observe similar increases in their sleep over the period. Additionally, although no historical information is available from other ways of measuring sleep duration, sleep estimates derived from accelerometry (Biobank, Willets, Hollowell, Aslett, Holmes, & Doherty, 2017) and the Munich Chronotype Questionnaire (MCTQ, T. Roenneberg, personal communication in March 2018) are added for 2015.

**Figure 3:**
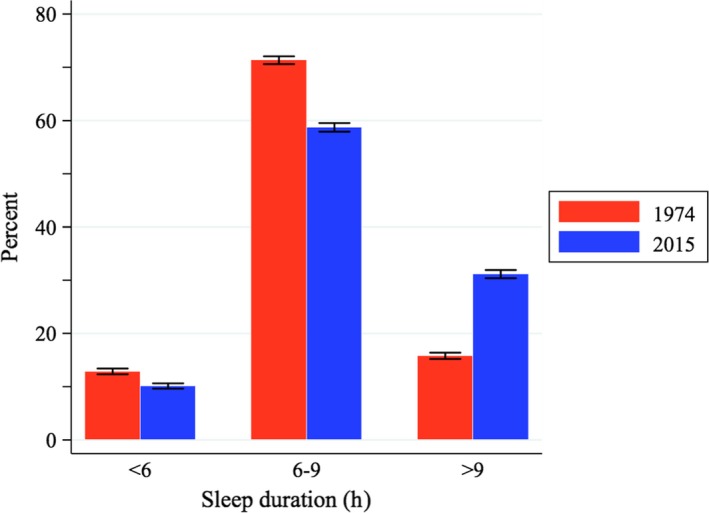
Change in sleep distribution in the UK between 1974 and 2015. The figure shows the prevalence of *short sleepers*(<6 hr), “*normal*” sleepers (6–9 hr) and *long sleepers*(>9) in 1974 and 2015. 95% confidence intervals are also shown. The *p*‐values of all changes shown are <0.001

The distribution of sleep time has shifted to the right (see Figure 3), making the sleep distribution more heterogeneous and increasing the prevalence of long sleepers by 15 percentage points (*p*‐value < 0.001). Short sleeping, however, has experienced only a mild decline of approximately 2.7 percentage points over the period (p‐value < 0.001). This change is driven by individuals aged below 65 years (see Table S3 in Supporting information, Appendix S3).

**Figure 4:**
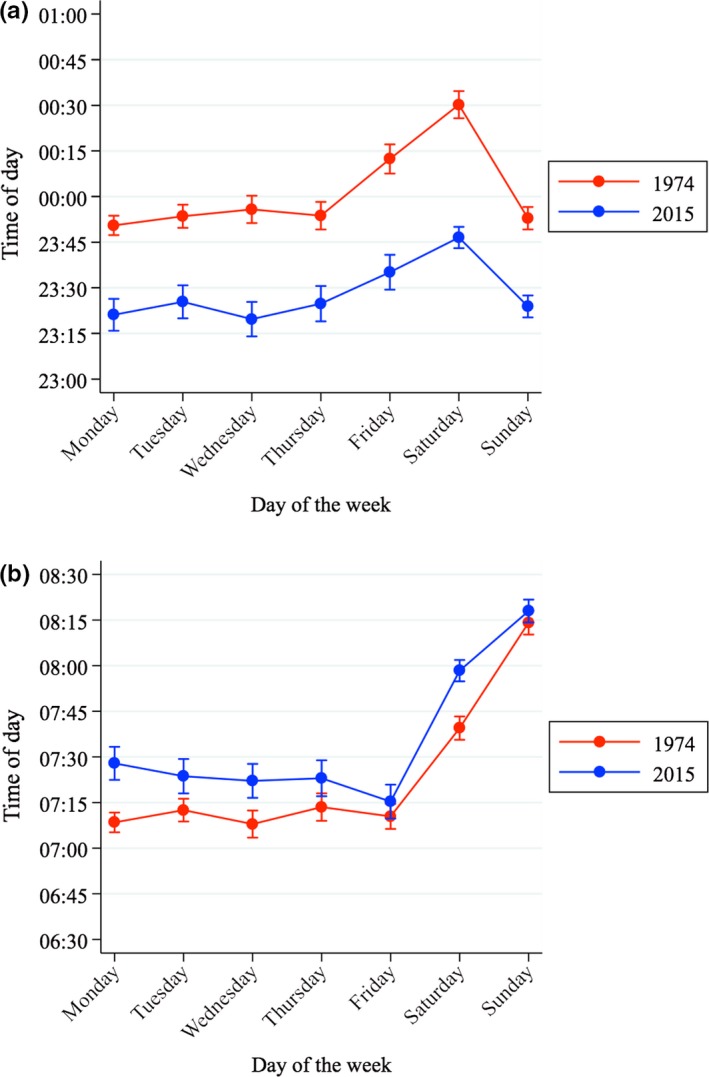
Sleep onset and sleep offset by day of the week in the UK in 1974 and 2015. (a) *Sleep onset*is the time at which the individual falls asleep in the evening. (b) *Sleep offset* is the time at which the individual wakes up

Figure 4 shows the changes in sleep onset and offset. Sleep onset happens ~30 min earlier in 2015 than in 1974, with the advance being a bit larger on Friday and Saturday nights, whereas sleep offset is delayed by ~15 min across the week.

### 3.2 Social jetlag

**Figure 5:**
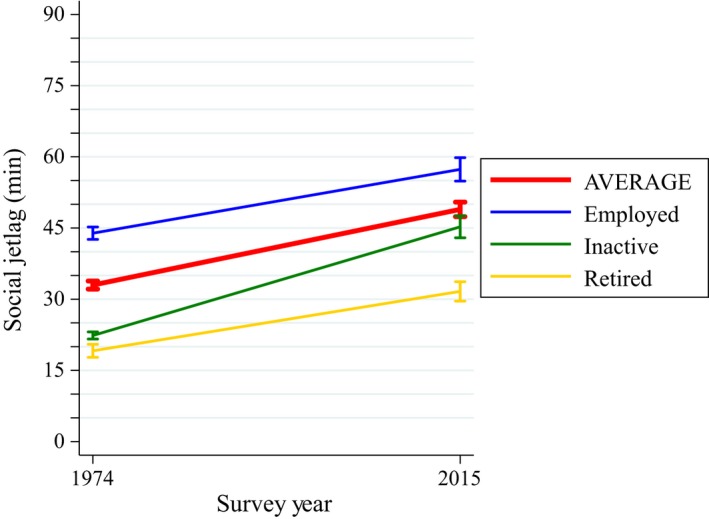
Change in social jetlag by employment status in the UK between 1974 and 2015. Social jetlag is defined following Jankowski's correction (Jankowski, 2017), which removes the sleep‐debt effect and captures only the effects of the biological/social time misalignment. *Jetlag = |Onset on free days − Onset on workdays|*for individuals with longer sleep and later (or equal) sleep onset on free days compared to workdays, *Jetlag = |Offset on free − Offset on workdays|*for individuals with longer sleep and earlier (or equal) sleep offset on workdays compared to free days, and *Jetlag *= *|Midsleep on free days − Midsleep on workdays|*for other sleep types. Weekdays and weekends are used instead of workdays and free days so that jetlag can be obtained for the entire population and not just for individuals in employment. Social jetlag increases by 16 min on average, by 13 min for the employed, by 23 min for the inactive and by 13 min for retired individuals (all *p*‐values < 0.001)

Social jetlag went up by 16 min on average (*p*‐value < 0.001) between 1974 and 2015 (Figure 5). Employed and retired individuals experience the smallest increase over the period (13 min, *p*‐value < 0.001), and the inactive the greatest with 23 min (*p*‐value < 0.001). Table S3 in the Supporting information, Appendix S3, shows the change in social jetlag based on gender and age as well.

### 3.3 Work and sleep

**Figure 6:**
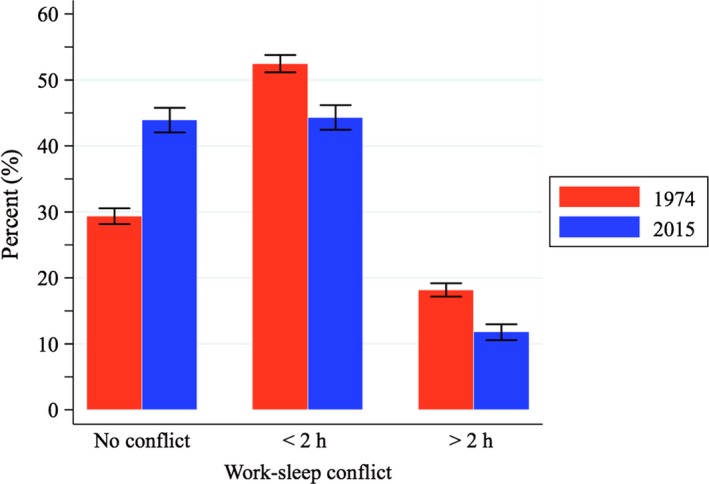
Change in work–sleep conflict in the UK between 1974 and 2015. Work–sleep conflict is defined as the amount of work that takes place within each individual's ideal sleep window (the 10 hr surrounding midsleep on free days). All the categories of conflict (conflict >0) become less popular in 2015, whereas the prevalence of “no conflict” increases by almost 15 percentage points. All these changes are statistically significant, as shown by the non‐overlapping confidence intervals (all *p*‐values < 0.001)

Since 1974 work time has changed significantly, becoming more heterogeneous both in terms of duration and timing. For instance, in 1974, the central 50% of the distribution (the difference between the 75th and 25th percentiles) in terms of daily work duration was found between 6.5 and 9.5 hr, whereas in 2015, the interval comprising that central 50% of the workforce had expanded by 1½ hr. The timing of work has also become more heterogeneous: in 1974, 50% of the most typical workers started work between 07:30 and 08:30 hours, whereas in 2015, the central 50% of the workers started work between 07:15 and 09:30 hours. To find out how these changes in work time relate to sleep in the population, Figure 6 explores the work–sleep conflict. There was a large work–sleep conflict in 1974 and there still is one in 2015, but it has declined substantively over the period, by 14.5 pp (*p*‐value < 0.001), from 70.6% in 1974 to 56.1% in 2015. Table S5 in the Supporting information, Appendix S3, shows how work–sleep conflict has gone down for men and women alike and also for all age groups, although slightly more for those aged 18–34 years than for middle‐aged people. The reduction in work–sleep conflict is partly explained by the increase in sleep duration and partly by the change to more sleep‐friendly work schedules.

## 4 DISCUSSION

It is widely believed that sleep deprivation is on the rise. However, the evidence for this is rather weak because it is based upon studies using the answer to a question like the following: On average, how many hours of sleep do you get in a 24‐hr period? Habitual sleep questions to measure sleep duration, even when they ask about workdays and non‐workdays separately, have been found to be imprecise when compared with actigraphy (Girschik et al., 2012; Lauderdale et al., 2008). Diaries have been shown to fare well when compared with camera recordings of subjects' daily activities (Gershuny et al., 2017), and existing diary evidence suggests time in bed has increased or stayed the same in most places (Bin, Marshall, & Glozier, 2012, 2013; Hoyos et al., 2015). This paper is, to our knowledge, the first one to investigate changes in actual sleep time in the UK.

The analysis shows that sleep has increased in the UK over the last four decades by 45 min on average, and a similar increase is found for individuals in employment. This increase is driven by both an earlier sleep onset and later sleep offset in 2015 compared with 1974. Although social jetlag (Wittmann et al., 2006) has increased over this period, direct work–sleep conflict has declined by almost 15 percentage points. These findings are hard to reconcile with the idea of an increasing sleep deprivation epidemic within the working population of the UK.

Between 1974 and 2015 the labour market in the UK became much more heterogeneous, both in terms of work duration and work timing. Such changes could be detrimental for people's sleep if they were imposed on workers. If, however, the increased heterogeneity is the result of worker demands, the effects could be beneficial. Given the variety in chronotypes, the increased variety in work start time could be due to individuals' choice. Although employer's demands may have contributed to the changes in the timing of work, individual requests are likely to have played an important role as well. Between 1974 and 2015 legislation was passed in the UK that enabled workers to reduce their work hours and to work whenever was more convenient for them, such as the Flexible Working Act of 2003 (not to mention the increase in self‐employment). A greater proportion of the population is now engaged in professional work that less often requires the employee's presence in the office at rigid times, and it is often possible to work anytime and anywhere. Hence, there is more opportunity to enhance sleep.

Despite the general improvements in sleep duration and work–sleep conflict, the data also show that there is a substantial part of the population (approximately 10%) that sleeps less than 6 hr a night (Table S3 in Supporting information, Appendix S3), 7% of the population experiences a social jetlag greater than 2 hr (not shown), and 56% experience work–sleep conflict (Table S5 in Supporting information, Appendix S3). There is further room for improvement in sleep in the UK, and maybe today's greater awareness of these sleep problems explains the view that sleep problems are on the rise.

A few limitations of the study should be considered. First, time‐use diaries are not an objective sleep measure and, as illustrated in Figure 2, different approaches for estimating sleep time in 2015 result in quite different estimates. This discrepancy also highlights the need for more user‐friendly technology to measure sleep in the real population (e.g. Debener, Emkes, Vos, & Bleichner, 2015). However, without putting too much weight on the absolute estimates of sleep time, the change as assessed with an identical methodology across time should be unbiased.

Second, although this study focuses on sleep duration, it is not clear that duration is the best or the only parameter to describe the “sleep health” of a population. The quality of sleep should be considered as well, especially because recent studies found an increase in sleep complaints and different measures of sleep quality over the last decades (e.g. Calem et al., 2012 for the UK; Pallesen, Sivertsen, Nordhus, & Bjorvatn, 2014 for Norway or Santos‐Silva et al., 2010 for the city of Sao Paulo). It is however, not entirely clear in some cases whether the apparent increase in these symptoms is not just the result of an increase in diagnosis. It is also possible that the greater report of sleep complaints is the result of raised awareness about our sleep and its importance for our health. More studies on this topic are clearly needed.

Sleep health might also relate to sleep timing (Carr et al., 2018) or the unequal allocation of sleep throughout the week (Wittmann et al., 2006). It is often suggested that any sleep variability is undesirable. However, experiencing some social jetlag may be acceptable too, or even natural. If we consider, for example, that retired individuals experience an average social jetlag of 32 min, half the level of individuals in employment but still well above zero, then we should allow for the possibility of some variability being part of a healthy sleep behaviour. Very recent evidence on sleep allocation throughout the week and mortality, gives support to the former idea. Åkerstedt et al. (2018) found that short weekday sleep is not a risk factor for mortality if it is combined with a medium or long weekend sleep. Similar observations have been made for obesity in children (Wing, Li, Li, Zhang, & Kong, 2009) and hypertension in adults (Hwangbo, Kim, Chu, Yun, & Yang, 2013).

Given that the length of sleep time has gone up in the UK and possibly in other places too, it becomes necessary to ask why, and future research should try to provide an answer. A first possibility is that the increase in sleep is comparable to luxury goods, the demand for which increases as societies become wealthier. If sleep is a pleasurable activity we could expect the time dedicated to it to increase. A second possibility is that the increase in sleep is just a by‐product of the increased time in bed resulting from the expansion of portable screens, which has in turn made us spend more time lying down and thereby creates more opportunities for sleep. A third possibility is that the increase in sleep is the result of an increased need for sleep. It is often suggested that the modern world is one where we work long hours and have little time for reflection. In that context it seems plausible to argue that more tiring days leave us with a greater need for sleep. Greater need for sleep could also come from a deterioration of sleep quality. Arguably, we could be substituting quality with quantity.

Finally, we may consider the possibility that the increase in sleep is the result of a natural tendency towards optimal sleep levels. What optimal sleep is, is still one of the great unknowns in sleep research (Roenneberg, 2013) and as a consequence current sleep recommendations (Hirshkowitz et al., 2015) are quite vague. Hence, we do not really know if current sleep levels in the UK, at around 8 hr per day, are closer to the optimal than the 1970s (7 hr and 20 min), as both fall within what is now considered the “normal” sleep window. If optimal sleep for the majority was 8 hr, economic growth and the rise in living standards in general would have simply enabled a greater part of the population to achieve optimal levels, just as more people can now afford to have a healthy diet than in the 1970s.

## CONFLICTS OF INTEREST

The authors declare no conflicts of interest.

## AUTHOR CONTRIBUTIONS

All authors contributed to the conception and design of the study. Data collection was carried out by JG and the analysis of the data by JL, with input from MD, JG and RF. JL wrote the first draft of the manuscript and all authors approved the final version.
