# Supplementary material for: Sleep differences in the UK between 1974 and 2015: Insights from detailed time diaries
Source: J Sleep Res. 2018 Sep 10;28(1):e12753. doi: 10.1111/jsr.12753 (PMC6378586; doi:10.1111/jsr.12753)
Supplement: Supplementary file 1 [file JSR-28-na-s001.doc]

APPENDIX 1. SLEEP IMPUTATION IN THE 1974/5 TIME USE STUDY

Three hours of the night are missing in the 1974/5 survey (2:00-5:00 am), and the 2014/5 survey was used to impute minutes of sleep in those three hours in a very non-controversial way. First, diaries from both surveys are divided into four groups depending on whether the diarist was asleep or awake at 2 and 5 am. Secondly, diaries from the 2015 survey are used to see how much on average do individuals in the different groups sleep between 2-5 am. Thirdly, those values are used to impute the missing sleep in 1974/5. While the proportions of the population in each of the groups are allowed to vary and do indeed vary between 1974 and 2015 (see Table 6), we consider it is reasonable to assume that the amount of sleep within each of those groups remains similar, and that is the assumption made in the manuscript. However, this is only an assumption, and in order to test the influence of such an assumption, we also added the most conservative (and unlikely) assumption, which is that people were always asleep between 2 and 5 am. Under this assumption, average sleep duration would still have increased over the period by 32 minutes (p-value<0.001)

Table 6. Sleep imputation between 2 and 5 am in 1974.

| Diaries defined by wakefulness at 2 and 5 am | 1974/5 | | 2014/5 | |
| --- | --- | --- | --- | --- |
|  | % of diaries | Imputed minutes of sleep between 2-5 am | % of diaries | Known average minutes of sleep between 2-5 am |
| 1. Asleep at 2 and 5 am | 88.5 | 177.2 | 83.2 | 177.2 |
| 2. Asleep at 2 and awake at 5 | 4.5 | 146.8 | 7.7 | 146.8 |
| 3. Awake at 2 and asleep at 5 | 5.6 | 106.3 | 7.1 | 106.3 |
| 4. Awake at 2 and 5 | 1.4 | 41.1 | 2.1 | 41.1 |
| Total | 100 | 169.9 | 100 | 167.1 |
