# Supplementary material for: Sleep differences in the UK between 1974 and 2015: Insights from detailed time diaries
Source: J Sleep Res. 2018 Sep 10;28(1):e12753. doi: 10.1111/jsr.12753 (PMC6378586; doi:10.1111/jsr.12753)
Supplement: Supplementary file 2 [file JSR-28-na-s002.doc]

APPENDIX 2. MEASURING TIME IN BED NOT ASLEEP IN THE SLEEP ESTIMATES

In order to properly remove time in bed not asleep from the sleep measure, not only does the code of “time in bed not asleep” need to exist – as it does in the two surveys used in this study, but diarists have to share the details of their falling asleep experience in their diaries. This appendix aims at showing that the majority of diaries do so and that it is therefore possible to estimate actual sleep time with sufficient precision.

In 1974/5 and 2014/5, 67.5% and 47.5% of diaries reported some time in bed not asleep (TIBNA>0), and 27.8 and 14.6% did not report any TIBNA (TIBNA=0) either because there was nothing to report, or it was too short to be reported. We know the this, because these diarists that did not report TIBNA at a given moment, did report it at some other point in the day, or in the other diary they fill in. Then, at least 95% and 62% of diaries in 1974/5 and 2014/5 respectively, did report their non-sleeping in bed (either TIBNA>0 or TIBNA=0). For the remaining diaries with no TIBNA reported (5% and 38% in 1974 and 2015 respectively), we cannot really know if that is because they do not experience TIBNA or because they fail to report. Hence some assumptions need to be made in order to provide an estimate.

Figure 6 shows the estimate of actual sleep time in 1974/5 and 2014/5, defined by midpoint of the interval that we describe next. The confidence interval is created by making two extreme assumptions about the possible values of TIBNA for this last group of diaries. The lower bound results from assuming that diaries with unknown TIBNA experience as much TIBNA as those who actually report positive values of TIBNA, and the upper bound from assuming that those diaries experience no TIBNA at all. The resulting “confidence intervals” are sufficiently narrow as to be certain of what has happened with sleep time.

To conclude, actual sleep time can be estimated with sufficient precision because not only did the surveys include activity schemes that allowed coding actual sleep and TIBNA separately, but also because in 1974/5 – the survey with longest interval and therefore the most prone to miss TIBNA, gave specific instructions to diarists to report it. The data collector (BBC) wanted to know what people were doing at each time to better programme their content and time in bed *before* and *after* night sleep was of particular interest. Therefore, specific instructions were given to diarists to identify clearly time in bed awake, and most diarists did so. This is why the reporting of TIBNA is done more often in 1974/5 than in the most recent survey.

Figure 6. Upper and lower bounds for actual sleep time in 1974/5 and 2014/5.
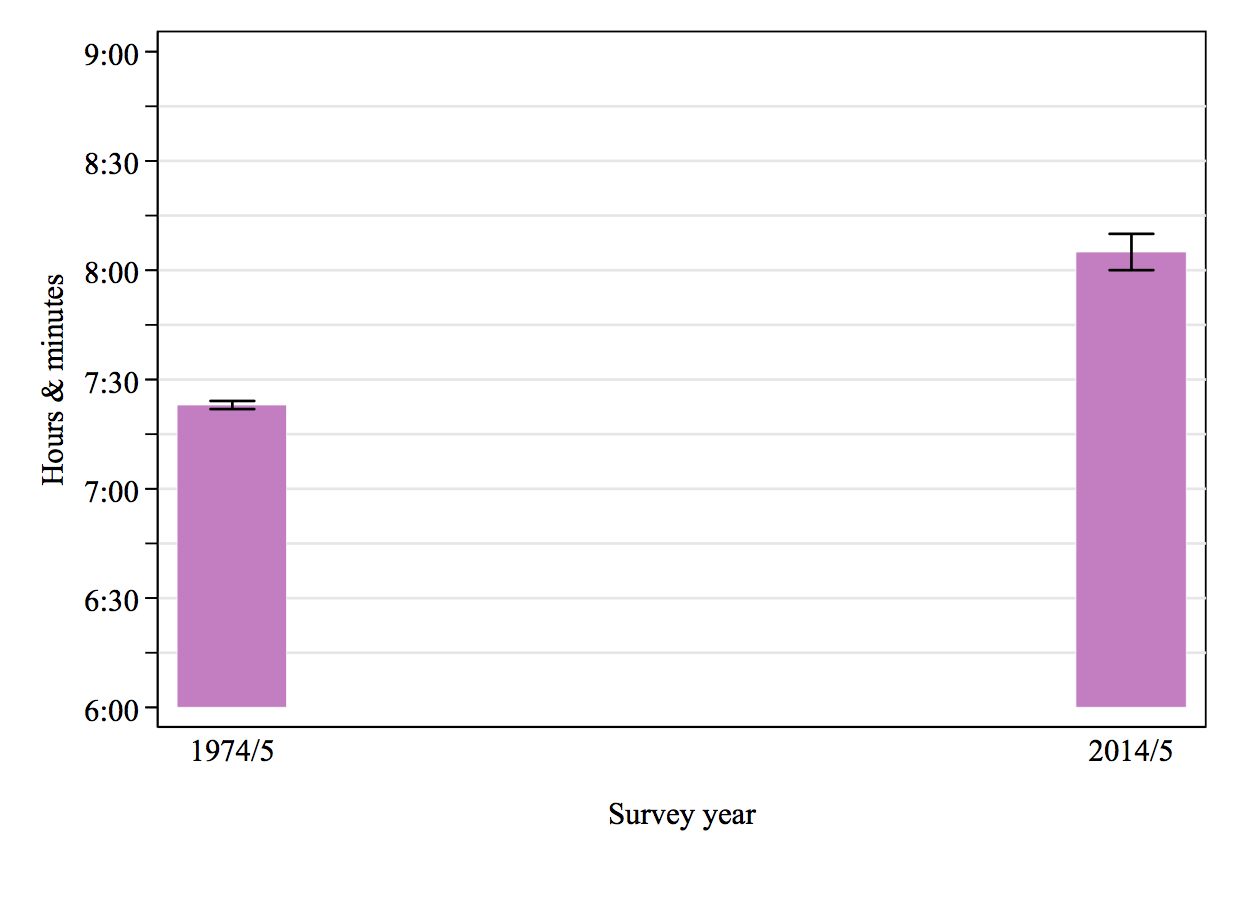


The upper bound comes from assuming that diaries with unknown time in bed not asleep experience none of it, and the lower bound from assuming that they experience as much of it as those who report it. The reason why the distance between the upper and lower bound is much narrower in 1974/5 than in 2014/5 is the much higher compliance in reporting time in bed not asleep as a result of survey instructions to that effect.
