# Supplementary material for: Sleep differences in the UK between 1974 and 2015: Insights from detailed time diaries
Source: J Sleep Res. 2018 Sep 10;28(1):e12753. doi: 10.1111/jsr.12753 (PMC6378586; doi:10.1111/jsr.12753)
Supplement: Supplementary file 3 [file JSR-28-na-s003.doc]

APPENDIX 3. EXTRA TABLES

Table 2. Sleep duration across sex, age, employment status, and type of day in 1974/5 and 2014/5.

|  | 1974/5 (minutes) | 2014/5 (minutes) | Change (minutes) | P-value |
| --- | --- | --- | --- | --- |
| *Sex* | | | | |
| Men | 441 | 482 | 41 | 0.000 |
| Women | 445 | 488 | 43 | 0.000 |
| *Age* | | | | |
| 18-34 | 452 | 509 | 57 | 0.000 |
| 35-64 | 431 | 472 | 41 | 0.000 |
| 65+ | 459 | 486 | 27 | 0.000 |
| *Employment status* | | | | |
| Employed | 434 | 478 | 44 | 0.000 |
| Inactive | 445 | 503 | 58 | 0.000 |
| Retired | 459 | 486 | 27 | 0.000 |
| *Type of day* | | | | |
| Weekday | 433 | 476 | 43 | 0.000 |
| Weekend | 468 | 510 | 42 | 0.000 |
|  |  |  |  |  |
| Total | 443 | 485 | 42 | 0.000 |

Table 2 shows sleep duration in the UK in 1974/5 and 2014/5 across sex, age, employment status, and type of day. Sleep duration includes day sleeping but excludes non-sleeping in bed such as sleep onset latency. The unemployed are included in the inactive as they were too few to be analysed on its own. Columns 1 and 2 show average sleep duration in 1974/5 and 2014/5. Column 3 shows the change, and the last column shows the p-value of an unpaired t-test comparing the two means.

Table 3. Prevalence of short sleeping (<6 hours) across sex, age, employment status and type of day in the UK in 1974/5 and 2014/5.

|  | 1974/5  (%) | 2014/5  (%) | Change (percentage points) | P-value |
| --- | --- | --- | --- | --- |
| *Sex* | | | | |
| Men | 13.9 | 10.6 | -3.3 | 0.000 |
| Women | 11.8 | 9.6 | -2.2 | 0.000 |
|  |  |  |  |  |
| *Age* | | | | |
| 18-34 | 13.7 | 8.5 | -5.2 | 0.000 |
| 35-64 | 14.3 | 11.7 | -2.6 | 0.000 |
| 65+ | 9.4 | 8.7 | -0.7 | 0.238 |
|  |  |  |  |  |
| *Employment status* | | | | |
| Employed | 14.9 | 11.0 | -3.9 | 0.000 |
| Inactive | 11.9 | 9.5 | -2.4 | 0.006 |
| Retired | 9.4 | 8.7 | -0.7 | 0.238 |
|  |  |  |  |  |
| *Type of day* | | | | |
| Weekday | 12.9 | 10.6 | -2.3 | 0.000 |
| Weekend | 12.7 | 8.9 | -3.8 | 0.000 |
|  |  |  |  |  |
| Total | 12.9 | 10.1 | -2.8 | 0.000 |

The table shows the prevalence of short sleeping (defined as sleep duration <6 hours) and its change in the UK in 1974/5 and 2014/5 across sex, age, employment status, and type of day. The first two columns show the prevalence of short sleeping in 1974/5 and 2014/5 respectively. The third column shows the change in short sleeping, and the last columns gives the p-value of an unpaired t-test comparing the two means.

Table 4. Social jetlag across sex, age, employment status in the UK in 1974/5 and 2014/5

|  | 1974/5 (minutes) | 2014/5 (minutes) | Change (minutes) | P-value |
| --- | --- | --- | --- | --- |
| *Sex* | | | | |
| Men | 40 | 54 | 14 | 0.000 |
| Women | 26 | 44 | 18 | 0.000 |
|  |  |  |  |  |
| *Age* | | | | |
| 18-34 | 47 | 60 | 13 | 0.000 |
| 35-64 | 34 | 50 | 16 | 0.000 |
| 65+ | 19 | 32 | 13 | 0.000 |
|  |  |  |  |  |
| *Employment status* | | | | |
| Employed | 44 | 57 | 13 | 0.000 |
| Inactive | 22 | 45 | 23 | 0.000 |
| Retired | 19 | 32 | 13 | 0.000 |
|  |  |  |  |  |
| Total | 33 | 49 | 16 | 0.000 |

Table 4 shows average social jetlag across employment status and age. Columns 1 and 2 show social jetlag (in minutes) for 1974/5 and 2014/5. The third column show the change (minutes) and the last column contains the p-value of an unpaired t-test comparing the two means. Social jetlag is defined following Jankowski’s correction (Jankowski 2017) that removes the sleep-debt effect and, capture only the effects of the biological/social time misalignment. *Jetlag=|Onset on free days – Onset on work days|* for individuals with longer sleep and later (or equal) sleep onset on free days compared to workdays, *Jetlag* *= |Offset on free – Offset on work days|* for individuals with longer sleep and earlier (or equal) sleep offset on workdays compared to free days, and finally, *Jetlag* = *|Midsleep on free days – Midsleep on work days|* for other sleep types. Weekdays and weekends are used instead of work and free days so that jetlag can be obtained for the entire population and not just for individuals in employment.

Table 5. Prevalence of work-sleep conflict across sex and age in the UK in 1074/5 and 2014/5

|  | 1974/5  (%) | 2014/5  (%) | Change  (percentage points) | P-value |
| --- | --- | --- | --- | --- |
| *Sex* | | | | |
| Men | 80 | 67 | -13 | 0.000 |
| Women | 58 | 46 | -12 | 0.000 |
|  |  |  |  |  |
| *Age* | | | | |
| 18-34 | 78 | 60 | -18 | 0.000 |
| 35-64 | 69 | 56 | -13 | 0.000 |
|  |  |  |  |  |
| Total | 72 | 57 | -15 | 0.000 |

Table 5 shows work-sleep conflict in the UK across sex and age. The first two columns show its prevalence in 1974/5 and 2015 (in %). The third column shows the change and the last one shows the p-value of an unpaired t-test comparing the two means. Work-sleep conflict can only be obtained for those in employment and that is why the age groups do not include those aged 65+. Work-sleep conflict is defined the amount of work within each individual’s ideal sleep window. Each person’s ideal sleep window is defined as the 10 hours surrounding midsleep on free days. Work time information is obtained from the diaries.
